# How ‘Cracks’ in Canada’s Public Services System Manifested as Moral (Di)Stress or Resilience for Emergency Management Personnel During COVID-19: A Critical Realist Study

**DOI:** 10.3390/ijerph23050604

**Published:** 2026-05-02

**Authors:** Andrew Schembri, Doris Yuet Lan Leung, Aaida Mamuji, Mac Osa Osazuwa-Peters, Charlotte T. Lee

**Affiliations:** 1 Daphne Cockwell School of Nursing, Toronto Metropolitan University, Toronto, ON M5B 2K3, Canada; andrew.schembri@torontomu.ca (A.S.); lee.charlotte@torontomu.ca (C.T.L.); 2 School of Nursing, The Hong Kong Polytechnic University, Hong Kong, China; 3 Disaster & Emergency Management, Faculty of Liberal Arts & Professional Studies, York University, Toronto, ON M3J 1P3, Canada; amamuji@yorku.ca (A.M.); mosazuwa@yorku.ca (M.O.O.-P.)

**Keywords:** emergency management, COVID-19, moral (di)stress, moral resilience, organizational resilience, structural distress, structural stigma, qualitative

## Abstract

**Highlights:**

**Public health relevance—How does this work relate to a public health issue?**

- Organizational resilience is an individual and collective resource to maintain operations and stability during public-sector crises.
- The detrimental impact of emergency management workers’ moral distress or injury eroded the resilience of emergency and public service systems during COVID-19.

**Public health significance—Why is this work of significance to public health?**

- An expanded model of moral stress is needed to address the structural components that generate individual moral distress or moral resilience.
- The study introduces a new model of systemic factors shaping moral stress revealed for Emergency Management Personnel (EMP) during, and in the aftermath of the COVID-19 pandemic.

**Public health implications—What are the key implications or messages for practitioners, policy makers and/or researchers in public health?**

- Privilege and positional authority shapes Emergency Management Personnel’s capacity and influence on operational realities.
- Upstream factors need to be addressed for Emergency Management Personnel to better enable their navigating structural competency between organizations and their operational requirements.

**Abstract:**

Organizations ought to demonstrate a responsibility for conditions that reduce moral stress and enhance moral resilience for their employees. No literature to date has explored how Emergency Management Personnel (EMP) experience both moral stress and distress [(di)stress], building up to stigma during health crises, given their role in emergency management operations. This study draws from a primary study of EMP, including frontline and first responders and those in leadership, who reported structural stigma during the COVID-19 pandemic. Our research question was, In what ways did structural stigma shape the moral landscape of emergency management practice during COVID-19? This qualitative study draws on the paradigm of critical realism to conduct thematic analysis. Interviews and focus groups were collected in 2024 from a total of 23 participants in the Greater Toronto Area, Canada. Participants represented EMP across emergency and public service sectors. System-level stressors revealed disruptions or “cracks” from an overwhelmed public services system. In sum, systemic “cracks” gave rise to organizational mechanisms designed to compensate for system failures, inadvertently propagating structural stigma. At times these mechanisms generated moral distress and/or resilience, through simultaneously expanding and limiting EMP’s responsibility and agency. The authors suggest that EMP build their leadership capacity to enhance skills of structural competency.

## 1. Introduction

During the COVID-19 pandemic, Canada experienced unprecedented health system challenges as rapid policy shifts, evolving public health directives, and widespread uncertainty disrupted routine operations across the country [1]. Indeed, the rapid spread of the COVID-19 virus revealed both long-standing structural gaps across health and emergency management systems [1,2,3]. Hence, the demands of the system exposed vulnerabilities in national and provincial emergency response capacities [1,2,3]. Central to the management of these pressures were Emergency Management Personnel (EMP), who were tasked to maintain the public’s safety [4].

EMP required coordination and collaboration between different political sectors, including public health, municipal services, the community, and federal emergency bodies; yet pre-pandemic evidence suggests that only a portion of Canadian emergency leaders in the health sector had received formal training or access to multi-agency cooperation and implementation necessary for large-scale health crises [5]. While this study situates COVID-19 within the context of a health crisis, it is important to distinguish between public health systems and emergency management systems.

EMP do not primarily deliver healthcare; rather, they operate at the intersection of governance, coordination, logistics, and implementation, translating public health and political directives into operational practice [4]. For example, in Canada, the increase in flooding and wildfires require EMP to coordinate multi-agency responses and operationalize public health and governmental directives to maintain response and safety in their communities [4]. Within this broader context, COVID-19 intensified demands on public service system leaders, contributing to heightened workloads for workers, fear of infection, and operational stress [1]. During COVID-19, emergency management was mobilized not simply to support healthcare delivery, but to address governance failures, inter-organizational coordination gaps, and prolonged uncertainty that exceeded the remit of public health alone [5]. As system pressures persisted, this brought to the forefront how organizational structures, policies, and practices heightened moral stress in the individuals embedded within it [6]. This distinction is critical, as EMP’s moral stress often emerged not from clinical decision-making, but from what Buchbinder et al. [6] suggest is from overstressed systems. Specifically, in Canada, EMP were tasked with enforcing, operationalizing, or justifying health policies over which they may have had limited influence or authority due to training, and EM resource variation [5].

In this study, systemic structures refer to institutionalized rules, policies, procedural logics, and material conditions (e.g., staffing, workload, role constraints) that organize work and routinized practice at the organizational level. These structures collectively constitute the institutional environment through which structural stigma is enacted and experienced [7]. Under the stress of the COVID-19 pandemic, systemic structures perpetuated structural stigma (i.e., entrenched institutional norms that reproduce unfair or unjust conditions), in reference to institutionalized rules, expectations, and procedures, unintentionally reinforcing inequities within work and operational environments [7,8].

During COVID-19, these system-level pressures did not remain abstract; it governed the lived experience of individuals, influencing their stress responses, ethical decision-making, and susceptibility to moral stress, moral distress or moral resilience during the prolonged crises [2,8]. Indeed, these constraints shaped one’s sense of professional integrity, as “*routine sources of stress were deeply embedded in the complex systems*” generating cumulative moral and emotional pressure [6], p. 15. In this context, such structural forces produce what Sukhera and colleagues [8] described as structural distress, in reference to a form of powerlessness. This experience was driven by organizational or policy decisions that contributed to mental exhaustion and compassion fatigue [8]. However, it is not yet known how systemic/structural components shape moral experience and the agency of individuals who cope with their moral (di)stress during a health crisis.

## 2. Literature Review: Structural Components and Agency Shaping Experience

### 2.1. The Structural Components

#### Structural Distress and Structural Stigma

Structural distress refers to the cumulative emotional and operational burden generated by navigating systems that reproduce disadvantages for groups, while expecting the same groups to uphold ethical standards without adequate support [8]. As a result, structural distress is best understood as a product of systemic/organizational forces that affect the individual workers’ sense of professional integrity over time, shaping both how they experience moral challenges and the degree to which they can enact agency to influence the conditions constraining them. 

In contrast, structural stigma is the systematic devaluation of certain groups through institutional policies, cultural norms, and power hierarchies, creating broader conditions under which structural distress emerges [7]. Structural distress reflects the chronic strain produced when workers must operate within environments shaped by inequitable resource allocation and inconsistent leadership. During COVID-19, Sukhera et al. [8] described this type of distress, not from isolated dilemmas, but from ongoing misalignments between individual needs and system-level responses.

### 2.2. Moral Experience

#### 2.2.1. Moral Stress, Moral Distress, Moral Injury, Moral Resilience, and Organizational Resilience

Cribb [9] differentiates the concept of moral stress from moral distress. Moral stress refers to the routine, pervasive pressures that arise when individuals working within complex systems persistently encounter processes that threaten their professional or ethical integrity [9]. This definition originated from the experiences of clinical staff, such as nurses and physicians, working in a healthcare environment [6,9]. Moral stress is not tied to a singular event, nor does it always produce emotional distress, but may result in positive consequences of learning one’s biases [6]. Indeed, moral stress reflects the deeply embedded structural and organizational barriers, shaped by institutional logic, policies, and everyday operational realities that repeatedly challenge workers’ sense of ethics in negative and positive ways [6].

Importantly, moral stress arises from the ongoing tension between what individuals believe they ought to do and what the system enables them to do [9]. When unaddressed, these routine system-level pressures can escalate into moral distress (i.e., feelings of powerlessness, shame, blame, or depression [6]) or, in extreme circumstances, the “residue” of distress can accumulate to create moral injury (i.e., lasting psychological injustice or harm to one’s identity [6]). Unlike moral distress, moral stress itself captures a broader, more consistent form of ethical strain that characterizes working within stressed or structurally constrained systems [6,10,11]. 

Moral distress has been widely studied in health ethics literature, but significant limitations remain in understanding the experience of moral distress outside of the clinical context. Bioethical literature often blurs the conceptual boundaries between moral stress and moral distress, which obscures the role of organizational and structural influences in shaping these experiences [6]. As Rushton [12] notes, it remains unclear how to build systems that consistently support ethical practice, as much of the existing literature emphasizes the emotional consequences of distress. Rather, Rushton [12] recommends exploring the process of how external system factors lead to internal conflict in individuals operating within them. Further, the literature tends to target individuals themselves to mitigate their moral distress, rather than changing systemic conditions [6,13]. Thus, the structural origins of what and how health system-level mechanisms influence components of individual (di)stress remain underexplored.

We argue that organizations ought to demonstrate responsibility for conditions that reduce moral stress and enhance moral resilience for their employees, beyond assumptions that existing rights and social structures sufficiently protect employees [14]. According to Nihlén Fahlquist [15], the primary responsibility of governments “is to make public-health decisions that create a balance between individual rights, on the one hand, and the health of the population, on the other” [15], p. 815. Hence, governments need to balance protecting the collective good and upholding individual ethical values [15]. To do this, organizations need to address the relational authority that forms their work contracts, in addition to the organizations’ fragmented decision-making processes [14]. In doing so, moral stress can motivate a search for organizational resilience [16].

We define organizational resilience as both an individual and collective resource driven by shared values and trust; to maintain operations and work through the fragmented social structures for stability during situations of uncertainty [17]. Navigating through this fragmentation can help groups react and recover from major disruptions, with minimal disturbance [17]. As part of resilience, individual and collective stress may incur. However, this can escalate to distress, eroding strategies that individuals use toward good stewardship of organizational resources [16]. Indeed, during COVID-19, leadership struggled with how to safeguard systems from adverse consequences of the social and public health measures themselves, when the individual autonomy of people was challenged [17]. 

Moral resilience is distinct, but part of general resilience; the former accounts for personal and relational integrity, self-regulation and awareness, adaptive “bouncing back,” moral efficacy, and self-stewardship [18]. Resilience is more widely understood as a resource for mitigating the negative impacts of burnout [18]. Beyond moral resilience being a protective resource over time, the recent literature states that it is the capacity of individuals to sustain, restore, or deepen their integrity in response to moral adversity, reframing the experiences of moral distress into opportunities for growth and forward movement [19,20,21]. Thus, it involves strengthening individual abilities to navigate ethical and moral challenges, within system-level supports and restrictions [19]. 

Organizations offer the “space” to promote or hinder a culture of ethical practice [14]. Thus, moral resilience is defined as positive adjustment to preserve one’s integrity during adversity, enabling one to align work with individual morality [12,18,19,20,21]. Epstein and Hurst [13] caution that positioning individuals to be solely responsible for their own moral resilience shifts responsibility away from organizational structures that contribute to moral stress. However, trust and solidarity are responsibilities of both the individual and the organizational governance [15]. 

#### 2.2.2. The Research Gap: The Experiences of Emergency Management Personnel

In the past 20 years, no literature to date has explored how structural components cause individual distress among EMP, given their crucial role in decisions, resource allocation, and operational coordination and implementation during health crises [5]. The existing literature about moral distress has focused predominantly on nurses and other clinicians [2], leaving a limited understanding of how system-level inequities, structural constraints, and organizational pressures shape the ethical experiences of those responsible for coordinating emergency responses [5]. Indeed, some evidence suggests that emergency management professionals within healthcare organizations acknowledge impediments to professionalization, including gaps in defining baseline professional competencies, standards for incident management, and standardized higher education [5]. However, these challenges are not uniform across emergency professions, as variations in historical development, education, and control over knowledge contribute to differing trajectories of professionalization across EMP [21]. Moreover, the lack of understanding of the role and experiences of EMP in relation to the pandemic response is essential to Canada’s health system [22], knowledge of which enables EMP to ethically navigate structural intersections while governing public health policy, emergency operations, and frontline realities [5].

Although the concepts of moral stress, moral distress, and moral resilience have been extensively examined within clinical and public health contexts, their application to emergency management requires careful conceptual adaptation. Unlike clinicians or public health practitioners, EMP are rarely the authors of health guidance; instead, they are positioned as intermediaries responsible for coordinating, implementing, and enforcing decisions made elsewhere [4]. As a result, moral distress among EMP is often rooted in responsibility without commensurate decision-making authority, limiting discretion within hierarchical command structures, and exposure to public scrutiny [23]. Therefore, they are implementing and held responsible for decisions they did not originate. This distinction underscores the need to examine moral experience among EMP as a function of governance and implementation dynamics rather than clinical ethics alone.

This study draws data from a primary study of Emergency Management Personnel (EMP), who possess a combination of higher education, specialized training in emergency response, and professional experience in related fields, such as nursing. In the primary study, EMP reported experiencing moral stress, moral distress, and moral injury during the COVID-19 pandemic [23]. The results from this study provided us with a lens for exploring how systemic structures shaped individual experiences in their reflexivity of learning post-COVID-19 pandemic.

## 3. Materials and Methods

### 3.1. Research Question

Our research question was, In what ways did structural stigma shape the moral landscape of emergency management practice during COVID-19?

### 3.2. Research Design

This qualitative study was guided by a critical realist paradigm, developed by the philosopher Bhaskar [24]. Critical realism acknowledges both experimental and interpretive lenses based on ontological positions concerning the nature of existence, alongside the view that epistemological claims (regarding how knowledge is produced) are inherently relative to the context [24]. Additionally, a realist approach assumes a connection between structural and individual factors, which helps make sense of causal effects through experiential, inferential, and dispositional themes [25]. This is why differences in outcomes occur for some individuals and not others [26]. Studies situated in social science, social psychology and health are the domains in which critical realism is best utilized [25,26]. This paradigm is well suited to exploring our phenomenon as it is concerned with the mechanisms across different levels of a stratified reality (i.e., post-positivistic objective and subjective) and can therefore determine theoretical factors and mechanisms that cause outcomes [24,25,26,27].

Critical realism is concerned with identifying how, why, for whom, and under what conditions different mechanisms bring about outcomes. It does so by distinguishing between three interconnected domains of reality [24,27]: The first is the “empirical,” which refers to what we can directly experience, such as our observations, thoughts, feelings, and accounts of events. The second is the “actual,” which encompasses the concrete events and interactions that occur, regardless of whether individuals are aware of them. The third is the “real,” which consists of the deeper causal powers and mechanisms that are often embedded in social structures and relationships [27]. The “real” refers to the inferential claims that give rise to both events and experiences, and which are partially revealed through what is not observed, in the “actual” (e.g., dispositions), and what is observed in the experiential or “empirical” domain [27].

### 3.3. Study Context: Canada and the Greater Toronto Area (GTA), Ontario, During COVID-19

Canada has a population of approximately 41.5 million people [28]. During the COVID-19 pandemic, those living in multigenerational households, racialized and Indigenous persons, and 2SLGBTQIA+ communities experienced the greatest burden of morbidity and mortality [29]. In Canada, the Greater Toronto Area (GTA) consists of 6.2 million people [30]. In 2021, the Census Report stated that 46.6% were immigrants, most of whom were women (53.9%), and born in the Philippines (10.3%), China (10.1%), and India (7.9%) [31]. Like the nation of Canada, multigenerational racialized households experienced a higher disease burden connected to having lower incomes, belonging to a visible minority, and not being able to receive government financial assistance [32]. Outlining this diversity and context is essential, as these inequities define the populations most at risk during disasters and, in turn, shape the context in which EMP operate and understand stigma within their communities. Given the role of EMP is in reducing a community’s vulnerabilities to disasters, we were compelled to explore how EMP addressed inherent public sector inequalities and structural distress in their communities.

### 3.4. Research Team’s Reflexivity

The research team was composed of five members. Two members conducted interviews and transcription preparation and contributed their expertise in disaster and emergency management (AM, MO). Three other members (AS, DL, CL) conducted data analysis, who possessed expertise in the paradigm of critical realism and nursing. Final preparation of the manuscript incorporated feedback from all research team members. The combination of these disciplinary perspectives and social identities informed how we approached, interpreted, and engaged with the data. Throughout the analytic process, the research team engaged in ongoing reflexive dialogue to consider how our professional roles, prior experiences, and underlying assumptions situated within emergency management structures influenced meaning-making. This intentional reflexive practice was used to enhance the study’s rigor and transparency.

### 3.5. Recruitment

While the primary study reports the methods [23] we summarize it briefly here. The research team employed purposive sampling, targeting EMP at different levels of government directly involved in the COVID-19 response. Recruitment was initiated through a call for participation email distributed via two main strategies: First, the research team contacted government and non-government institutions with relevant leadership personnel, including police and firefighters, emergency medical services, public health officials, members of the armed forces, and NGO/volunteer organizations. The email contained a brief description of the project, a link to the project’s website, and a participant recruitment flyer. Institutions were asked to circulate the call for participation internally and to designate an employee who acted as a contact person for the research team. Confirmation of circulation through the designated contact persons served as institutional permission to approach employees directly. The research team then connected with interested employees to determine eligibility, provided further information about the study, and obtained informed written consent, prior to scheduling data collection. Participants were also invited to recommend additional experts who met the study’s inclusion criteria as part of a snowball recruitment strategy.

Second, the research team sent an email to the Ontario Association of Emergency Managers (OAEM). The email included the project description, website link, and recruitment flyer, and requested that the OAEM designate a contact person and circulate the email to social media platforms. Interested members were asked to contact the research team directly. As in the first strategy, recruitment occurred through snowball sampling.

### 3.6. Data Collection

Data consisted of individual interviews and focus groups collected in 2024 for a total of 23 participants in the Greater Toronto Area, Canada. Saturation was reached when we had 23 participants, and therefore, recruitment ended. Participants represented EMP from a broad range of frontline and leadership roles across emergency and public service sectors. The interview guide was developed by authors of the primary study. Two research team members conducted all the interviews. Briefly, the interview guide asked: (1) In general what does COVID-19 stigma mean to you? (2) Did stigmatization impact your work or the people you serve? If so, how so? (3) Did you experience any stigma toward you as a person? If so, how so? (4) What would you do differently to ensure a better response to stigma-related issues (that we have discussed) in future emergencies? Interviews each lasted approximately 30 mins to 90 mins. Care was taken not to impose interview time parameters on participants, given their busy schedules.

### 3.7. Data Analysis

Data were analyzed using Fryer’s [32] four-phase approach following critical realist thematic analysis, which includes: (i) familiarization with the dataset; (ii) developing and refining codes; (iii) constructing and reviewing themes; and (iv) generating theoretical interpretations and final reporting. Under the guidance of the lead analyst (CL), two members of the research team (AS, DL) first immersed themselves in the material through repeated readings to develop early analytic impressions, note potential patterns, and identify questions arising from participants’ accounts.

To begin coding, AS and DL independently applied descriptive, empirical-level codes to approximately 20% of the interviews (n = 4). They then met to compare their coding decisions, discuss interpretations, and resolve any discrepancies. This early stage supported the development of a shared coding framework and enhanced analytic credibility.

As the researchers moved through additional transcripts, they iteratively shifted between data familiarization and code development, working across critical realism’s “empirical” and “actual” domains. Once consensus was reached on the initial coding structure, it was applied to the remaining interviews. Each transcript was coded independently by either AS or DL, with any new or uncertain codes discussed with a second team member to ensure interpretive plausibility and consistency. Through this iterative process, recurring patterns were identified and clustered into preliminary themes that reflected underlying mechanisms and structures at the level of the “actual” domain [33].

In the final phase, the team integrated inductive insights with deductive reasoning drawn from the literature on structural stigma, moral stress, and moral distress. This process of theoretical engagement enabled the researchers to posit deeper generative mechanisms operating at the level of the “real” and to articulate how structural conditions shaped participants’ experiences [33]. These interpretive decisions informed the development of the final thematic structure representing tendencies toward how EMP were positioned to experience structural distress, and differential moral outcomes.

### 3.8. Trustworthiness

To ensure analytic rigor, the research team adhered to Fryer’s [33] four criteria for evaluating the trustworthiness of critical realist qualitative work: credibility, plausibility, transferability, and utility. Credibility and plausibility were supported through ongoing comparison of codes between at least two researchers at each analytic stage, with discrepancies resolved through discussion and reflexive deliberation. Throughout the analysis, the team maintained detailed theoretical memos to document analytic decisions, reflexive insights, and the evolution of interpretive claims, providing a clear audit trail.

Peer debriefing occurred regularly with the lead analyst (CL) and the broader research team, allowing for interrogation of emerging themes and refinement of theoretical propositions. Transferability and utility were strengthened by grounding interpretations in rich participant quotations and by situating the results within the broader context of COVID-19 emergency management services. Finally, the reporting of methods and results aligns with recognized qualitative standards, including guidance from the EQUATOR network’s reporting recommendations for qualitative research, to support transparency and comprehensiveness [34].

### 3.9. Ethical Aspects

Ethical approval for the parent study from which this dataset was drawn was granted by the University Research Ethics Board (REB #: E2024-114). Written informed consent was obtained from all participants and reaffirmed prior to data collection. All materials were stored on encrypted, password-protected servers to maintain confidentiality. For this secondary analysis, only de-identified transcripts, prepared by members of the primary research team, were used. The authors conducting the secondary analysis did not participate in data collection and therefore engaged with anonymized transcripts only. In all reporting, participants are referred solely by interview numbers to protect individual identities and ensure confidentiality.

## 4. Results

### 4.1. Participant Characteristics

A total of 23 participants consisted of: emergency management officials (n = 9), fire service officers (n = 5), police service officers (n = 3), non-governmental organization employees (n = 3), public health officials (n = 2), and paramedics (n = 1). This range of professional backgrounds provided insight into both operational and policy-level dimensions of emergency response during the COVID-19 pandemic. While participants are collectively referred to as Emergency Management Personnel, they occupied distinct leadership roles across emergency management, public health, enforcement, and non-governmental sectors, each with different degrees of authority, visibility, and influence over pandemic decision-making. The diversity in their roles highlights the differing lenses through which they view the events of the COVID-19 pandemic. 

Participants were drawn from four organizational levels: municipal (n = 12), regional (n = 6), provincial (n = 2), and non-governmental organizations (n = 3). This multilevel representation captured perspectives across operational, tactical, and strategic domains of the emergency management system. It included both frontline responders and senior officials involved in coordination, planning, or high-level decision-making. The diversity of roles, institutional contexts, and leadership tiers allowed for a nuanced understanding of moral stress, structural distress, and structural stigma shaping decision-making, interpretation, and negotiation across different points in the emergency management hierarchy.

### 4.2. Thematic Results

**Figure 1:**
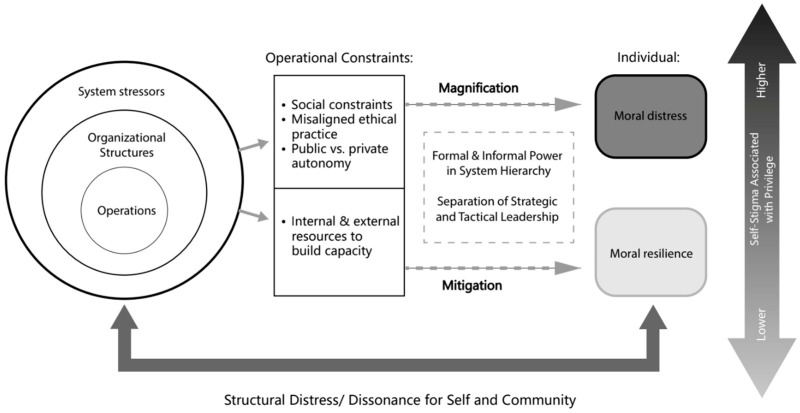
Structural factors shaping moral distress–resilience model.

Our thematic analysis generated the “*Structural Factors Shaping Moral Distress-Resilience Model*” (Figure 1), which illustrates how system stressors during a health crisis can result in EMP experiencing either moral distress or moral resilience. System-level stressors revealed disruptions or “cracks” from an overwhelmed healthcare system. System, organizational, and operational stressors were nested in layers, illustrating how each structural environment interacted to produce or alleviate moral distress. Operational constraints, including social norms, misaligned ethical practice, and the tension between public and private autonomy, are at the center of the system, highlighting the ways EMP were positioned at the point of enactment. Moral distress and moral resilience were magnified or mitigated, respectively, while varying levels of privilege shaped proximity to self-stigma and agency. We refer to privilege in reference to positional authority, access to information and proximity to impact decision making. In what follows, structural distress lies on a continuum experienced both individually and collectively, emphasizing how dissonance for self and community emerged throughout the pandemic response.

Figure 1 outlines mechanisms visually: system stressors, organizational structures, and operational constraints as nested and intertwined layers. These layers converged to influence whether stress was magnified (represented by the darker colors in the vertical self-stigma arrow), producing conditions in which participants experienced disruptions, and triggered moral stress. These factors created moral distress or opportunities for resilience. These two possible configurations form formal and informal power hierarchies, regulatory ambiguity, and separation between strategic and tactical leadership; all of which magnified or mitigated structural stigma and moral burden (represented by the dotted arrows). Conversely, a lower-pressure configuration with transparent communication, collaborative leadership, and access to internal and external resources supported participants in working through and “bouncing back” from stress. These two possible configurations reflected the broader continuum of which individuals experienced structural stigma, distress, and/or resilience, as it unfolded throughout the pandemic.

### 4.3. System Stressors

System-level stressors formed the outermost layer of the model and shaped how EMP experienced moral stress during the COVID-19 pandemic. Moral distress surfaced as participants navigated disruptions or “cracks” in the system: rapidly changing directives, misaligned ethical expectations, and significant tensions, often without the guidance or resources needed to respond effectively. These structural stressors accumulated over time, shaping how participants understood and performed their roles and responsibilities and the amount of agency they felt when responding to high-stakes decisions.

Participants repeatedly described how hierarchical, paramilitary structures within rapidly changing provincial directives created significant ambiguity at the operational level. The absence of unified guidance from provincial authorities placed EMP in the position of having to interpret, enact, and justify decisions without adequate information. As one participant reflected:

“*The implementation of closing of schools, closing of gyms*,*… The region did not make that decision… that’s all provincial directive. But when they passed it… province stayed silent on whether or not somebody who was vaccinated or not was able to reenter the workforce of those; that was a decision that the individual entity had to make…. It was not a yes or no type of question, you know, there’s legal there’s legal consequences to it. So, I thought that was kind of strange*”.

(Interview 16)

This excerpt illustrates the system-level bottlenecks that characterized the early pandemic response and the turbulence that EMP navigated as a result. Across interviews, EMP emphasized that decisions made at the provincial level lacked transparency and were often delivered without a corresponding framework to guide implementation. This created a sense of system unpreparedness and left municipalities without the support required to respond effectively. Participants described a culture in which information was controlled at the upper levels of government, reinforcing a top-down structure that privileged process over people and limited the ability of local emergency managers to anticipate or influence decisions. 

System stressors were further exacerbated by the absence of preparedness planning despite lessons from the 2002 global outbreak of Severe Acute Respiratory Syndrome (SARS). Participants consistently noted that lessons learned from response structures, resources, and communication pathways had not been meaningfully operationalized prior to COVID-19. For many EMP, these system-level stressors were intensified by expectations that emergency management structures would compensate for gaps in public health preparedness and political decision-making. However, these social structures were not designed or resourced for prolonged health governance. Consequently, EMP entered the pandemic already disadvantaged by bureaucratic constraints and fragmented system oversight, fueling early moral stress as they attempted to align their professional obligations with unclear and shifting directives. 

### 4.4. Organizational Stressors

Organizational-level dynamics further intensified participants’ moral stress by shaping how they interpreted, enacted, and responded to system directives. Participants described an organizational landscape characterized by blurred boundaries between strategic and tactical work. This context limited the sharing of information between units, and a pervasive sense of uncertainty around roles and responsibilities. As one participant articulated:

“*The overarching theme is, you know, you were not satisfying either side’s ideas 100% right, trying to strike that balance. And in those cases, you know, it’s not a matter of doing what’s best for the individuals or dictating to them what we think is best. It’s really an organizational perspective on what’s the global perspective on this, and what’s the best thing for us to do organizationally*”.

(Interview 8)

This excerpt captures a central tension within organizational stressors: the need to balance community expectations, internal mandates, and external political pressures, often without adequate clarity or direction for individual responsibility in their role. Participants described working within siloed structures where communication pathways were inconsistent and where critical information flowed unevenly across departments. This contributed to ambiguity around decision-making authority. It also impaired participants’ ability to build coherent shared mental models of risk. 

Organizational ambiguity was particularly acute for emergency managers tasked with operational coordination, who frequently navigated misalignment between public health guidance, political authority, and frontline realities without a clear mandate to reconcile these tensions.

Paramilitary cultural norms, including adherence to the chain-of-command and deference to higher-level directives, further constrained their agency. Participants’ agency was particularly restricted when frontline insights conflicted with strategies developed at higher levels. Many participants expressed a sense of organizational unpreparedness and emphasized how the pandemic exposed a lack of education and role standardization within the field of emergency management [5]. This limited recognition of EMP expertise reinforced structural barriers and created contextual conditions in which moral stress accumulated as workers navigated responsibilities that they were expected to fulfill without sufficient authority or support. In sum, systemic “cracks” gave rise to organizational mechanisms designed to compensate for system failures, inadvertently propagating structural stigma. At times these mechanisms generated moral distress and/or resilience, through simultaneously expanding and limiting EMP’s responsibility and agency.

### 4.5. Operations

Operational dynamics represented the level at which structural and organizational stressors became most tangible, often transforming moral stress into acute moral distress. EMP were frequently placed in situations where they were responsible for enforcing policies that generated conflict between public health directives and individual autonomy, exposing them to heightened community stigma. One participant described such an encounter:

“*And these are all women, okay, racialized women, yeah, and not in my nursing home, but in all nursing homes, right? But in my nursing home, it was a little bit worse, so it was more horrible. But, you know, and all of them, I could not believe it… I’m like, somebody’s got to stop this, because we’re going to lose the little staff that we have*”.

(Interview 11)

Direct encounters, such as the one described (above) highlight how EMP operated at the interface of structural directives and operational responses, shouldering the emotional and ethical burden of enforcing contested practices. Participants described these encounters as moments in which misaligned ethical practice and public–private autonomy collided, intensifying the experience of stigma and positioning them as targets of frustration from both community members and those in leadership roles.

Despite these challenges, participants also described operational practices that mitigated distress and created the conditions necessary for moral resilience. These practices centered on collaborative decision-making and the integration of frontline perspectives into strategic planning. This also included intentional dialogue that bridged the gap between strategic and tactical roles. As one participant noted:

“*When we are pitching a new idea, I kind of tap them to say, like, you guys are on, like, start picking this apart and tell us where we’re wrong. Tell us where we need to go… we have an Operational Advisory Committee… frontline paramedics, where we bring these ideas to them too, and say, we’re going to do this. What do you think? Pick it apart*”.

(Interview 17)

Leadership that fostered shared understanding, redistributed moral and cognitive burdens, and strengthened operational cohesion mitigated participants’ moral stress. By legitimizing frontline expertise and enabling two-way communication, these practices reduced uncertainty and counteracted the amplification of moral distress that occurred in more rigid or hierarchical operational environments.

### 4.6. Structural Distress Across Varying Levels of Privilege and Agency

The final theme captures how varying levels of privilege, influence, and positional authority shaped EMP’s experiences of moral distress. Distress was not evenly distributed; instead, it varied according to employees’ proximity to leadership structures, public scrutiny, and responsibility for high-stakes decisions. Some participants described a form of identity-based distress rooted in the burden of representing their profession during contentious phases of the pandemic. As one participant stated:

“*I guess the stigma I felt, not personally, was more as the XXX role in that how we move as an organization and manage as an organization, is going to have an impact on our whole profession… It was more keeping my head on that way, versus not letting it affect me personally*”.

(Interview 6)

This excerpt reflected how structural conditions produced dissonance between personal values and professional expectations, compelling workers to manage reputational and political consequences beyond their individual control. As moral stress intensified, disruptions or “cracks” formed to challenge EMP’s responsibility to cope with their moral distress, alone or with others, including peers. Indeed, across the system level, participants described how rapidly evolving directives, unclear jurisdictional authority, and limited access to information created conditions in which moral distress was nearly unavoidable. 

Participants also described how moral distress prompted compensatory actions, including: informal peer support (i.e., “*I did it unofficially to try to check in with each of the folks*”—Interview 20); formal consultation (i.e., “*We actually did an assessment of all our staff, a survey, on how we were doing responding to COVID.*”—Interview 3); and educational strategies aimed at reducing harm (i.e., “*We were consulting with our wellness people. We have staff psychologists.*”—Interview 10). When EMP were enabled to strengthen community relationships, they used their privilege to lower their moral (di)stress and generate moral resilience. For example, one participant explained:

“*We worked with our community groups before we publish the information, but you know, I think it was; it was felt to be stigmatizing, especially when you don’t have a reason behind it… we didn’t share it*”.

(Interview 4)

This excerpt represented a response to mitigate structural stigma and navigate the tension between transparency and community protection. These experiences revealed how structural and organizational forces intersected with individual moral agency, shaping the pathways through which moral stress progressed toward either distress (“cracks” in the system) or resilience.

In sum, the “Structural Stressors Shaping Distress-Resilience Model” positioned EMP to experience a continuum of structural distress/dissonance moderated by privilege, influence, and access to decision-making power. Altogether, this model demonstrates how structural conditions created varying opportunities for EMP to resist or internalize their moral burden during the COVID-19 crisis.

## 5. Discussion

During the COVID-19 health crisis, system-level stressors were revealed in structural distress, which we refer to as disruptions, representing “cracks” in the health system. These structural stressors shaped EMP’s experience of moral stress, and for some, moral distress or moral resilience. In brief, our model theoretically suggests multilevel factors through which structural stigma shaped participants’ moral experiences. In other words, while individual coping and personal characteristics influence how moral distress is experienced, these factors are shaped and influenced by participants’ positioning within a paramilitary governance system. Yet moral distress was not experienced uniformly; it varied according to participants’ situated agency and influence on decision-making, that is, their privilege.

Structural stigma functioned differently across this continuum, at times exacerbating their sense of vulnerability and/or their professional reputation. Participants with higher positional authority (privilege) described distress connected to representing their profession and navigating political scrutiny. In contrast, other middle management staff experienced distress stemming from direct public encounters and limited structural supports. This gradient reflects a continuum of privilege in which varying degrees of agency shaped how moral stress was interpreted, internalized, or resisted. Moreover, these results underscore the importance of understanding moral distress as a relational and structural phenomenon, in which individual responses to challenging circumstances are shaped and conditioned by broader systemic factors. In sum, our model advances existing understandings of moral (di)stress by highlighting the interplay of systemic, organizational, and operational environments and how these conditions collectively shape participants’ agency, as well as structural constraints.

While literature confirms that the Canadian healthcare system was ill-equipped to manage the pressures of COVID-19 [1,3,35], our findings illustrate how organizational mechanisms were dynamic, in that they contributed to moral distress by simultaneously expanding participants’ responsibility and limiting their agency. Participants described blurred boundaries between strategic and tactical responsibilities, inconsistent communication pathways, and cultural norms that limited access to information. In this sense, moral distress was not merely an emotional outcome; it was a structurally produced ethical experience, which appeared “inevitable” [9]. Structural stigma displayed participants’ misalignment that positioned them between political authority and public accountability. As a result, participants routinely faced an ethical burden of justifying policies they neither shaped nor fully understood. This reflected the idea of “powerlessness,” a key indicator of moral distress [6]. 

Like the literature on “middle managers” in education [36] and in nursing [37], at the level of operations, EMP encountered the most tangible experiences of moral distress at the level of operations. Aligned to a scoping review by Hertelendy et al. [38], moral agency is connected to belonging to a team, to enact professional standards; indeed, this assumes an interdependency on how one is positioned with others in the hierarchy to hold power. Thus, participants also described operational contexts in which moral distress was mitigated, particularly when leadership facilitated open dialogue, drew on their clinical expertise, and created processes through which participants could meaningfully contribute to decision-making. In other words, one’s agency essentially required organizational leaders to appropriately distribute their moral burden to groups, enhance coherence between values and practice, and support their moral resilience through their relationships with community leaders [37].

This study’s model contributes to the broader literature by foregrounding the structural determinants of moral experience in emergency management work and offering a framework to understand how organizational and political conditions shape ethical outcomes during public health crises. By identifying mechanisms that mitigate and magnify moral (di)stress, this work provides a foundation for designing emergency response systems that support ethical practice, psychological well-being, and resilient relationships in future crises.

### 5.1. Implications

Given that distress was frequently amplified by inconsistent directives, limited information flow, and unclear operational jurisdiction, our study suggests that these indicators may be a way to recognize structural distress. Moreover, the paramilitary hierarchy, demonstrated in our study, revealed how dominant bias was internalized by groups and communicated to others. As such, our study results suggest that governing leadership needs to be more intentional, to encourage collaboration rather than exclude leadership of EMP across all health system levels. In comparison, Davis and Batcheller [39] argued that addressing moral distress requires access to a “resilience bundle.” This is in reference to organizationally supported leadership and infrastructure facilitating one’s agency to cope with their moral (di)stress [39]. As in our study, beyond personal strategies, a resilience bundle included access to: informal discussions with colleagues; structured/formal debriefings; educational activities to recognize moral distress and its consequences; and formal counseling, that is, employee assistance to address individual well-being [39]. EMP’s experiences revealed how privilege and positional authority shaped their influence on operational realities. 

As found in the previous literature about leadership’s role to support and mitigate moral distress [40], we suggest that EMP build their leadership capacity in work positioned between organizational policy and operational requirements, that is, to enable skills of “structural competency” [41]. Structural competency refers to learning that addresses upstream factors affecting individuals’ access to resources, to knowledge of how social structures impact groups, and to facilitating advocacy not only within organizations, but also in neighborhoods and cities [41]. Developing structural competency among EMP requires recognizing their distinct role in governance and implementation, rather than positioning emergency management as an extension of the healthcare workforce.

Finally, this study underscores an urgent need for additional research examining how system-level factors produce or alleviate moral (di)stress in emergency management work. Future work should explore how leadership structures, communication ecosystems, and organizational cultures can be redesigned to enhance moral resilience, particularly during prolonged emergencies.

### 5.2. Limitations

This study has several limitations that influence the transferability of its findings. First, the sample was drawn from a single large metropolitan region in Canada, where emergency response structures, population diversity, and inter-agency relationships may differ from those in rural or less resource-dense settings. Second, the timing of data collection multiple years into the COVID-19 pandemic may have influenced participants’ reflections, as experiences of acute stress may have evolved into more retrospective interpretations. Third, the interdependence of EMP with municipal, regional, provincial, and public health leadership created complex relational dynamics that may have intensified perceptions of structural stigma during the pandemic. As such, the degree to which similar dynamics exist in non-crisis conditions remains unclear. Additionally, because participants varied widely in positional authority, proximity to decision-making, and operational responsibilities, their experiences of moral stress and distress may reflect role-specific influences not fully captured in this analysis. Similarly, the analysis may not fully capture the experiences of individuals whose voices were absent or underrepresented in the sample, particularly those in less visible or lower-authority roles, which may influence interpretations when moving between the layers of critical realism. 

Future research should examine moral distress among EMP across different jurisdictions and governing stress-generating structures [6]. Furthermore, researchers could investigate potential moderating factors such as task interdependence, shared mental models, and organizational preparedness that may shape vulnerability to structural distress, as well as pathways beyond resilience to moral repair [6]. Longitudinal studies would also help clarify how experiences of moral stress evolve over time and how resilience may be strengthened within emergency management systems.

## 6. Conclusions

This study demonstrates that structural stigma, hierarchical decision-making, and fragmented communication systems were key mechanisms transforming moral stress into moral distress among Emergency Management Personnel during COVID-19. At the same time, collaborative leadership practices and inclusive operational structures created opportunities for moral resilience, illustrating that distress is not an inevitable outcome of moral stress but rather a product of surrounding system conditions.

Our findings underscore the need for structural reform that enables EMP to better manage ethical conflict with support and be responsive to the complex relational environment in which they operate. Building systems that support transparent information flow, shared decision-making, and recognition of EMP’s expertise is essential for repairing the cycle of structural distress, that is, the “cracks” or disruptions in health crises that have historically occurred across all levels of public service systems governance.

As health systems continue to face evolving public health threats, EMP and their governing leadership must recognize and address the ways in which structural forces create “cracks” in the system, and work to fill them through skills of structural competency. Supporting EMP through improved coordination, structurally informed leadership development, and system-level preparedness strategies will be critical not only for mitigating moral distress but also for fostering a more resilient, ethically grounded emergency response system.

## Data Availability

Data supporting this study are included within the article.
